# Attenuating Effects of Dieckol on Endothelial Cell Dysfunction via Modulation of Th17/Treg Balance in the Intestine and Aorta of Spontaneously Hypertensive Rats

**DOI:** 10.3390/antiox10020298

**Published:** 2021-02-16

**Authors:** Seyeon Oh, Minjung Shim, Myeongjoo Son, Ji Tae Jang, Kuk Hui Son, Kyunghee Byun

**Affiliations:** 1 Functional Cellular Networks Laboratory, Department of Medicine, Graduate School and Lee Gil Ya Cancer and Diabetes Institute, College of Medicine, Gachon University, Incheon 21999, Korea; seyeon8965@gmail.com (S.O.); dreamer1192@gmail.com (M.S.); mjson@gachon.ac.kr (M.S.); 2 Department of Anatomy & Cell Biology, Gachon University College of Medicine, Incheon 21936, Korea; 3 Aqua Green Technology Co., Ltd., Smart Bldg., Jeju Science Park, Cheomdan-ro, Jeju 63309, Korea; jtjang@aquagt.co.kr; 4 Department of Thoracic and Cardiovascular Surgery, Gachon University Gil Medical Center, Gachon University, Incheon 21565, Korea

**Keywords:** endothelial cell dysfunction, T helper 17, regulatory T cell, gut barrier, *Ecklonia cava*, dieckol

## Abstract

Disruptions of the Treg/Th17 cell balance and gut barrier function are associated with endothelial dysfunction. Dieckol (DK) obtained from *Ecklonia cava* and *E. cava* extract (ECE) decreases blood pressure by reducing inflammation; however, it has not been elucidated whether DK or ECE modulates the Treg/Th17 balance, changes the gut epithelial barrier, or decreases endothelial cell dysfunction. We evaluated the effects of ECE and DK on gut barrier and the Treg/Th17 balance in the intestine and aorta, with regard to endothelial dysfunction, using the spontaneously hypertensive rat (SHR) model. The level of Th17 cells increased and that of Treg cells decreased in the intestine of SHRs compared to normotensive Wistar Kyoto (WKY) rat. These changes were attenuated by ECE or DK treatment. Additionally, the serum IL-17A level increased in SHRs more than WKY; this was decreased by ECE or DK treatment. The level of Treg cells decreased and that of Th17 cells increased in the aorta of SHRs. These changes were attenuated by ECE or DK treatment. The NF-κB and IL-6 levels were increased in SHRs, but these changes were reversed by ECE or DK treatment. Endothelial cell dysfunction, which was evaluated using peNOS/eNOS, nitrate/nitrite ratio, and NADPH oxidase activity, increased in the aorta of SHRs, but was decreased by ECE or DK treatment. The Treg/Th17 balance in the intestine and aorta of SHRs was attenuated and endothelial cell dysfunction was attenuated through the Th17/NF-κB/IL-6 pathway by ECE or DK.

## 1. Introduction

As a physical barrier, the gut epithelial barrier, composed of epithelium, mucus lining, and junctional proteins, is involved in modulating the absorption of nutrients and limiting the passage of pathogens or unwanted molecules into the systemic circulation [1]. Structural changes of the gut epithelial barrier lead to the loss of barrier integrity, resulting in uncontrolled permeability of luminal content and allowing harmful substances into the systemic circulation [1]. Loss of intestinal barrier integrity is involved in development of inflammatory state in intestinal diseases and extraintestinal disease such as hypertension [2,3,4,5].

Studies have shown that gut permeability, which was evaluated by measuring fluorescein isothiocyanate (FITC)-dextran in the plasma, was increased in adult spontaneously hypertensive rat (SHR) compared with that in age-matched normotensive Wistar Kyoto (WKY) rat [5,6]. In addition, the number of goblet cells, which produce mucus, and the levels of gut junctional proteins such as occludin, tight junction protein 1, and claudin 4 were decreased in SHR compared with the levels in WKY rat [5,6]. These gut junctional proteins are essential for controlling epithelial barrier function, and expression of those proteins are changed in diseases that relate with increased permeability [7]. 

T helper 17 (Th17) cells are a unique CD4^+^ T helper subset that produces interleukin (IL)-17A, which aggravates tissue inflammation [8,9]. Under homeostatic conditions, Th17 cells, which are abundant in the lamina propria of the small intestine [10], are involved in the protection of mucosal surfaces against microbial pathogens [11,12]. However, when Th17 cells are converted into IL-17-producing T cells by IL-23 or IL-6, inflammation occurs [13].

IL-17A binds to IL-17 receptor A (IL-17RA) and activates various signal pathways related to inflammation, such as nuclear factor-κB (NF-κB) [14]. For NF-κB activation, IL-17RA associates with an adaptor protein, nuclear factor-kappa B activator 1 (ACT1)/ tumor necrosis factor receptor-associated factor 3 interacting protein 2 (TRAF3IP2) [15,16], and TNF receptor-associated factor 6 (TRAF6) [14]. IL-17A leads to upregulation of the cytosine-cytosine-adenosine-adenosine-thymidine (CCAAT)/enhancer-binding proteins (C/EBPs), such as C/EBPβ and C/EBPδ, which lead to increased IL-6 expression [17]. While Th17 cells induce inflammation by secreting IL-17A, regulatory T cells (Tregs), act as suppressors of inflammation by secreting the anti-inflammatory cytokine IL-10 [18,19]. 

Modulation of Th17/Tregs balance by increasing Treg cells restored tight junction of intestinal epithelial cell barrier in the inflammatory bowel disease [20]. It is known that imbalanced Th17/Treg cells are related with development of cardiovascular diseases such as hypertension [21]. Treg cells in the spleen of stroke-prone spontaneously hypertensive rats decreased compared with normotensive WKY rats, even before the onset of hypertension [21]. Th17/Tregs imbalance is related with plaque destabilization which induced acute coronary syndrome [22]. Endothelial dysfunction is the main pathophysiological factor underlying hypertension. Many clinical studies have shown a positive correlation between the plasma IL-17 level and endothelial dysfunction [23]. 

Serum/glucocorticoid-regulated kinase 1 (SGK1) is essential for regulating Th17/Treg balance [24]. IL-23 stabilized and reinforced Th17 cells to increase pathogenic effector action by increasing IL-23 receptor [24,25]. SGK1 is downstream of IL-23 receptor and involves the differentiation of Th17 cells by regulating expression of IL- 23 receptor [24]. Loss of SGK1 decreases the expression of IL-23 and reduces development of pathogenic Th17 cells [24]. In additional, transcription factor forkhead box P3 (Foxp3) is essential for differentiation of Treg cells [26]. Forkhead box protein O1 (FOXO1), serves as a placeholder of Foxp3 and stabilizes Foxp3 during Treg cell differentiation [27]. SGK1 induced nuclear exclusion of FOXO1 by phosphorylation and induced degradation [28,29]. Treg cell-specific deletion of SGK1 enhanced Treg cells by preventing FOXO1 nuclear exclusion and increased Foxp3 expression by binding to Foxp3 [30]. 

Meanwhile, dieckol (DK), a phlorotannin present in *Ecklonia cava*, is known to exert antihypertensive effects by inhibiting angiotensin 1-converting enzyme [31]. Previously, our group reported that *E. cava* extract (ECE) attenuated endothelial cell dysfunction by reducing the inflammation of perivascular fat tissue [32]. Although DK or ECE has attenuating effects on hypertension and endothelial cell dysfunction, it has not been determined whether DK or ECE modulates the Treg/Th17 balance in the intestine, leading to changes of the gut epithelial barrier related to endothelial cell dysfunction in hypertension. In the present study, using SHRs as the study model, we evaluated the effects of ECE and DK on gut barrier changes and changes of the Treg/Th17 balance in the intestine and aorta leading to endothelial dysfunction.

## 2. Materials and Methods

### 2.1. Preparation of ECE and Isolation of DK

*E. cava* was obtained from Aqua Green Technology Co., Ltd. (Jeju, South Korea). For extraction, *E. cava* was washed and air-dried at room temperature for 48 h, after which the blades were ground and 50% ethanol was added, followed by incubation at 85 °C for 12 h. ECE was filtered, concentrated, sterilized by heating to ≥85 °C for 40–60 min, and then spray-dried. DK, one of the representative phlorotannins present in ECE, was isolated using centrifugal partition chromatography (CPC). CPC was performed using a two-phase solvent system comprising water/ethyl acetate/methyl alcohol/n-hexane (7:7:3:2, *v/v/v/v*). The organic stationary phase was filled in the CPC column, followed by pumping of the mobile phase into the column in descending mode at the same flow rate used for separation (2 mL/min). We finally confirmed that the purity of the DK used in the study was 93.58% [33].

### 2.2. Hypertension Animal Model

Male SHRs (aged 8 weeks, *n* = 25, *n* = 5 for each of the 5 groups) and WKY rats (aged 8 weeks, *n* = 5) were obtained from Orient Bio (Seongnam, Republic of Korea) and housed at a constant temperature of approximately 23 °C, relative humidity of 50%, and a dark/light cycle of 12/12 h. The rats were acclimated to the conditions for 1 week. The rats were then randomly categorized into six groups. For 4 weeks, the rats were orally administered drinking water (WKY/water, *n* = 5 or SHR/water, *n* = 5), and SHRs were orally administered ECE (SHR/ECE50, 50 mg/kg/day, *n* = 5; SHR/ECE100, 100 mg/kg/day, *n* = 5; or SHR/ECE150, 150 mg/kg/day, *n* = 5) or DK (SHR/DK, 2.5 mg/kg/day, *n* = 5). At the end of the four-week study period, blood pressure of all rats was measured by using a noninvasive tail-cuff CODA system (Kent Scientific Corp., USA) [34,35]. After blood pressure measurement, rats were sacrificed. All animal experiments were performed with approval according to the ethical principles of the Institutional Animal Care and Use Committee of Gachon University (approval number: LCDI-2019-0121). All of the experiments were repeated three times per animal.

### 2.3. RNA Extraction and complementary DNA (cDNA) Synthesis

The rat intestine and aorta were homogenized in ice using a disposable pestle in 1 mL of RNisol (Takara, Tokyo, Japan), and homogenates were added to 0.2 mL of chloroform, mixed, and centrifuged at 12,000× *g* for 15 min at 4 °C. The aqueous phase was collected, placed in cleaned tubes, mixed with 0.5 mL of isopropanol, and centrifuged at the same conditions. The supernatant was discarded, leaving only the RNA pellet that was then washed with 70% ethanol and dissolved in 50 µL of diethyl pyrocarbonate-treated water. The isolated RNA was synthesized with cDNA using a Prime Script 1st strand cDNA Synthesis Kit according to the manufacturer’s instructions (Takara, Tokyo, Japan).

### 2.4. Quantitative Real-Time Polymerase Chain Reaction (qRT-PCR)

Quantitative real-time polymerase chain reaction (qRT-PCR) was performed using cDNA synthesis by the CFX384 TouchTM Real-Time PCR detection system. Two hundred nanograms of cDNA, 5 μL of SYBR premix (Takrara, Tokyo, Japan), 0.4 μM forward and reverse primers (listed in Table S1) were mixed, then threshold cycle numbers were determined using CFX Manager^TM^ software [36]. 

### 2.5. Immunohistochemistry (3,3-diaminobenzidine: DAB)

Tissue blocks of paraffin-embedded intestine and aorta were cut into 7 µm-thick sections, placed on a coated slide, and dried at 45 °C for 24 h. Slides were deparaffinized and incubated in normal animal serum to block antibody nonspecific binding, and then incubated with primary antibodies (listed in Table S2) at 4 °C, followed by three additional rinses with phosphate-buffered saline (PBS). Slides were then treated with biotinylated secondary antibodies using an ABC kit (Vector Laboratories, Burlingame, CA, USA), incubated for 1 h with secondary antibody solution, and rinsed three times with PBS. Slides were left to react with DAB substrate for up to 15 min, followed by mounting with a cover slip and DPX mounting solution (Sigma-Aldrich, St. Louis, MO, USA) [37,38,39]. Images were obtained using a light microscope (Olympus, Tokyo, Japan) and the intensity of the brown color was quantified using ImageJ software (NIH, Bethesda, MD, USA) [32]. 

### 2.6. Histology Analysis 

#### 2.6.1. Staining with Periodic acid–Schiff (PAS)

Staining with PAS was used to determine the intestinal mucosa with goblet cells in absorptive columnar epithelium. Deparaffined intestine tissue sections were stained with using the PAS staining kit (BBC Biochemical, McKinney, TX, USA). The intestinal tissue slides were oxidized in 1% periodic acid solution (BBC Biochemical, McKinney, TX, USA) for 5 min, rinsed in distilled water, and placed in Schiff reagent (BBC Biochemical, McKinney, TX, USA) for 20 min at room temperature. Nuclei were stained with Harris’ hematoxylin (DAKO, Glostrup, Denmark), and the slides were cover-slipped using a mounting medium and observed under an optical microscope (Olympus Optical Co., Nagano, Japan). The number of PAS-positive goblet cells per μm^2^ were counted by using ImageJ (NIH, Bethesda, MD, USA) [40]. Morphometrical analysis was conducted in a blinded manner and three operators conducted at least three replicates of each analysis.****

#### 2.6.2. Hematoxylin and Eosin (H&E) Staining

H&E staining was used for determining the intestinal pathologic changes including villi length and tunica muscularis thickness. The intestinal tissue slides were incubated with hematoxylin (DAKO, Glostrup, Denmark) for 1 min, rinsed in distilled water for 10 min, and placed in eosin Y solution (Sigma-Aldrich, USA) for 1 min at room temperature. Nuclei were detected with blue color and cytoplasm was detected with light pink, and the completed slides observed under an optical microscope (Olympus Optical Co., Nagano, Japan). The villi length and tunica muscularis thickness were measured by ImageJ (NIH, Bethesda, MD, USA) [41]. Histological analyses were conducted in a blinded manner and three operators conducted at least three replicates of each analysis. 

### 2.7. Indirect Enzyme-Linked Immunosorbent Assay (ELISA)

To measure the serum IL-17A and IL-10 levels, 1 mL of blood was centrifuged and incubated in serum separator tubes (Becton Dickinson, USA) for 30 min. Samples were then centrifuged at 2000× *g* for 10 min and the supernatant was transferred to a new tube. The transparent serum specimens obtained were stored in a freezer at −80 °C.

The 96-well microplates were coated with anti-IL-17A and anti-IL-10 antibodies diluted in 100 nM carbonate and bicarbonate mixed buffer, adjusted to pH 9.6, and incubated overnight at 4 °C. The microplates were then washed with PBS containing 0.1% Triton X-100 (TPBS). The remaining protein-binding sites were then blocked using 5% skim milk for 6 h at room temperature [42]. After washing with PBS, the serum samples were distributed to each well and incubated overnight at 4 °C. Each well was rinsed with TPBS and then incubated for 4 h at room temperature with a peroxidase-conjugated secondary antibody. Tetramethylbenzidine solution was added, followed by incubation for 15–20 min at room temperature. Sulfuric acid (2N) was used as a stop solution. Optical density was measured at a wavelength of 450 nm using a microplate reader (Spectra Max Plus; Molecular Devices, San Jose, CA, USA).

Nitrate/nitrite levels (780001; Caymanchem, Ann Arbor, MI, USA) and NADP/NADPH^+^ ratio (ab65349; Abcam, Cambridge, England) in the aorta of each group were determined using the appropriate kit, in accordance with the manufacturer’s instructions. 

### 2.8. Statistical Analysis

Nonparametric tests were performed in this study. The Kruskal–Wallis test was used to determine the significance of differences among the WKY/water, SHR/water, and SHR/ECE150 groups. If significant difference was confirmed by Kruskal–Wallis, multiple comparison was performed with the Mann–Whitney U test. Results were presented as mean ± SD, and statistical significance was accepted as follows: *, versus WKY/water; $, versus SHR/water; and #, versus SHR/ECE150. Statistical analysis was performed using SPSS version 22 (IBM Corporation, Armonk, NY, USA).

## 3. Results

### 3.1. ECE and DK Decreased Th17 Cell Levels, Increased Tregs Cell Levels, and Decreased IL-17A Levels in the Intestine of SHR

**Figure 1:**
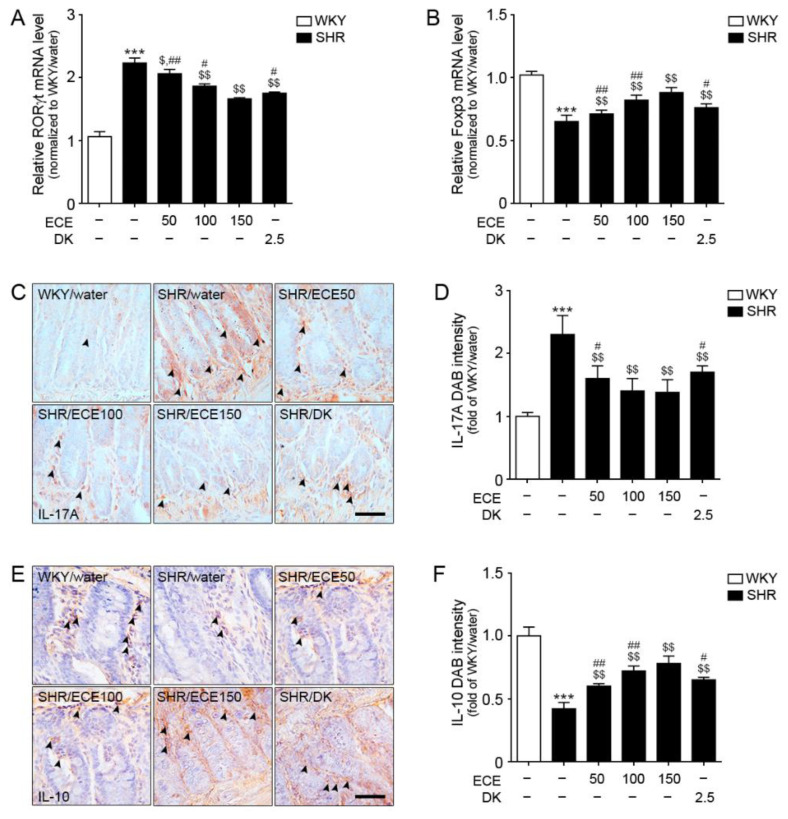
Regulatory effects of DK and ECE on Th17 and Treg cell levels in the intestine of SHRs. (**A**) In intestinal tissues, RORγt mRNA levels were increased by SHR/water. The addition of ECE and DK decreased the RORγt and Foxp3 mRNA levels. (**B**) The Foxp3 mRNA level in intestinal tissues was decreased following SHR/water and increased by ECE or DK treatment. (**C**,**D**) The IL-17A expression level in intestinal tissues was increased following SHR/water and decreased following ECE or DK treatment. (**E**,**F**) The IL-10 expression level in intestinal tissues was decreased following SHR/water and increased following ECE or DK treatment. For each of the 6 groups, *n* = 5. Scale bar = 100 µm. ***, *p* < 0.001, vs. WKY/water; $, *p* < 0.05 and $$, *p* < 0.01, vs. SHR/water; #, *p* < 0.05 and ##, *p* < 0.01, vs. SHR/ECE150 (Mann–Whitney U test). DK, dieckol; ECE, *Ecklonia* *cava* extract; Foxp3, forkhead box P3; IL-10, interleukin-10; IL-17A, interleukin-17A; RORγt, retinoic-acid-receptor-related orphan nuclear receptor gamma t; SHR, spontaneously hypertensive rat; WKY, Wistar Kyoto rat.

The mRNA expression level of a Th17 cell marker (RORγt) in the intestine of SHRs was higher than that in WKY rats, but it was decreased following treatment with either ECE or DK (Figure 1A). The decreasing effect was most prominent in the 150 mg/kg ECE treatment. The mRNA expression level of a Treg marker (Foxp3) in the intestine of SHRs was significantly lower than that in WKY rats (Figure 1B), but it was significantly increased following the administration of either ECE or DK. The increasing effect was most prominent in the 150 mg/kg ECE treatment.

The expression level of IL-17A in the intestine of SHRs was significantly higher than that in WKY rats (Figure 1C,D). It was significantly decreased following the administration of either ECE or DK. The decreasing effect was most prominent in the 100 and 150 mg/kg ECE treatment. The expression level of IL-10 in the intestine of SHRs was significantly lower than that in WKY rats (Figure 1E,F). It was significantly increased following the administration of either ECE or DK. The increasing effect was most prominent in the 150 mg/kg ECE treatment.

### 3.2. ECE and DK Treatment Attenuated Destruction of Gut Barrier Integrity and Decreased SGK1 Expression in the Intestine of SHRs

**Figure 2:**
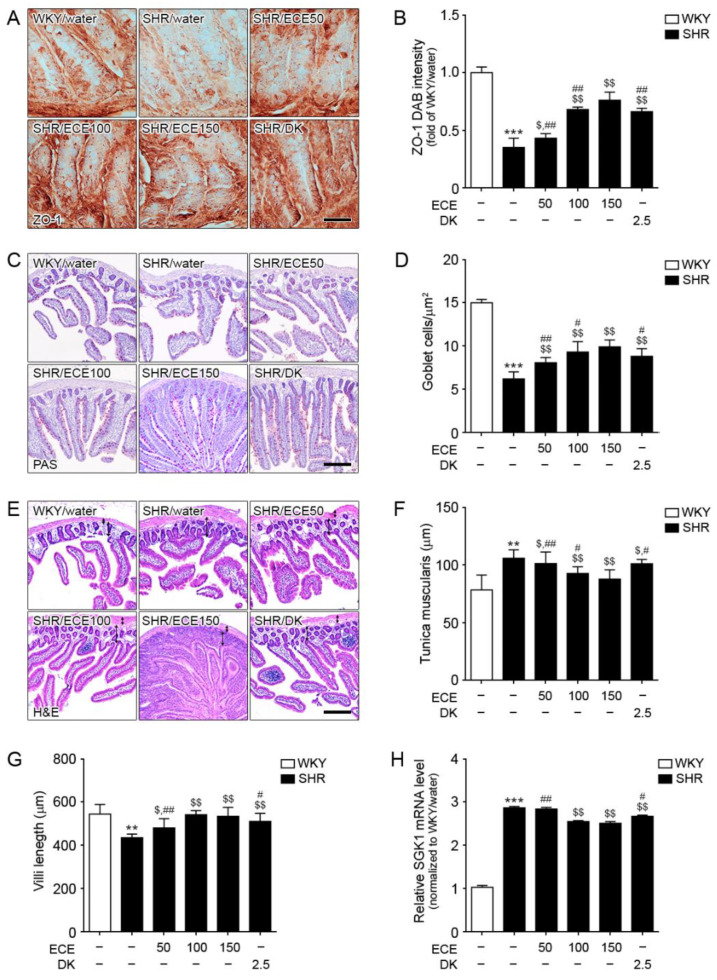
Regulatory effects of DK and ECE on the destruction of gut barrier integrity and SGK1 level in the intestine of SHRs. (**A**,**B**) The ZO-1 expression level in intestinal tissues was decreased following SHR/water and increased following ECE or DK treatment. (**C**,**D**) The number of goblet cells in intestinal tissues was decreased following SHR/water and increased following ECE or DK treatment. (**E**–**G**) The tunica muscularis thickness and villi length in intestinal tissue were measured by H&E staining. (**H**) In intestinal tissues, SGK1 mRNA levels were increased following SHR/water. The addition of ECE and DK decreased the SGK1 mRNA level. For each of the 6 groups, *n* = 5. Scale bar = 100 µm. **, *p* < 0.01 and ***, *p* < 0.001, vs. WKY/water; $, *p* < 0.05 and $$, *p* < 0.01, vs. SHR/water; #, *p* < 0.05 and ##, *p* < 0.01, vs. SHR/ECE150 (Mann–Whitney U test). DK, dieckol; ECE, *Ecklonia* *cava* extract; HE, hematoxylin and eosin staining; PAS, periodic acid–Schiff; SGK1, serine/threonine-protein kinase; SHR, spontaneously hypertensive rat; WKY, Wistar Kyoto rat; ZO-1, zonula occludens-1.

The expression level of zonula occludens-1 (ZO-1), a tight junction protein of the gut barrier, was evaluated to confirm gut barrier integrity (Figure 2A,B). The expression level of ZO-1 in the intestine of SHRs was significantly decreased compared with that in WKY rats. It was significantly increased following the administration of either ECE or DK. The increasing effect was most prominent in the 150 mg/kg ECE treatment. The mRNA expression level of occludin 1 in the intestine of SHRs was significantly decreased compared with that in WKY rats (Figure S1). It was significantly increased following the administration of either ECE or DK. The increasing effect was most prominent in the 150 mg/kg ECE treatment.

The number of goblet cells in the PAS-stained intestine was decreased in SHRs compared with that in WKY rats (Figure 2C,D). It was significantly increased following the administration of either ECE or DK. The increasing effect was most prominent in the 150 mg/kg ECE treatment.

The thickness of tunica muscularis was increased in SHRs compared with that in WKY rats (Figure 2E,F). It was significantly decreased following the administration of either ECE or DK. The decreasing effect was most prominent in the 150 mg/kg ECE treatment. The villi length of was decreased in SHRs compared with that in WKY rats (Figure 2E,G). It was significantly increased following the administration of either ECE or DK. DK attenuated endothelial cell dysfunction via modulation of Th17/Treg balance in the intestine and aorta of spontaneously hypertensive rats

The increasing effect was most prominent in the 100 and 150 mg/kg ECE treatment. The mRNA expression level of SGK1 in the intestine of SHRs was significantly increased, which was in turn significantly decreased following the administration of 100 and 150 mg/kg ECE and DK (Figure 2H). The decreasing effect was most prominent in the 100 and 150 mg/kg ECE treatments. 

### 3.3. ECE and DK Reduced Serum IL-17A Levels and Increased Serum IL-10 Levels in SHRs

The serum level of IL-17A in SHRs was significantly higher than that in WKY rats. This level was decreased following the administration of either ECE or DK). The decreasing effect was most prominent in the 150 mg/kg ECE treatment (Figure S2A). 

The serum level of IL-10 in SHRs was significantly lower than that in WKY rats, but it was significantly increased following the administration of either ECE or DK. The increasing effect was most prominent in the 150 mg/kg ECE treatment (Figure S2B).

### 3.4. ECE and DK Decreased Th17 Cell Levels and Increased Tregs Cell Levels in the Aorta of SHRs

**Figure 3:**
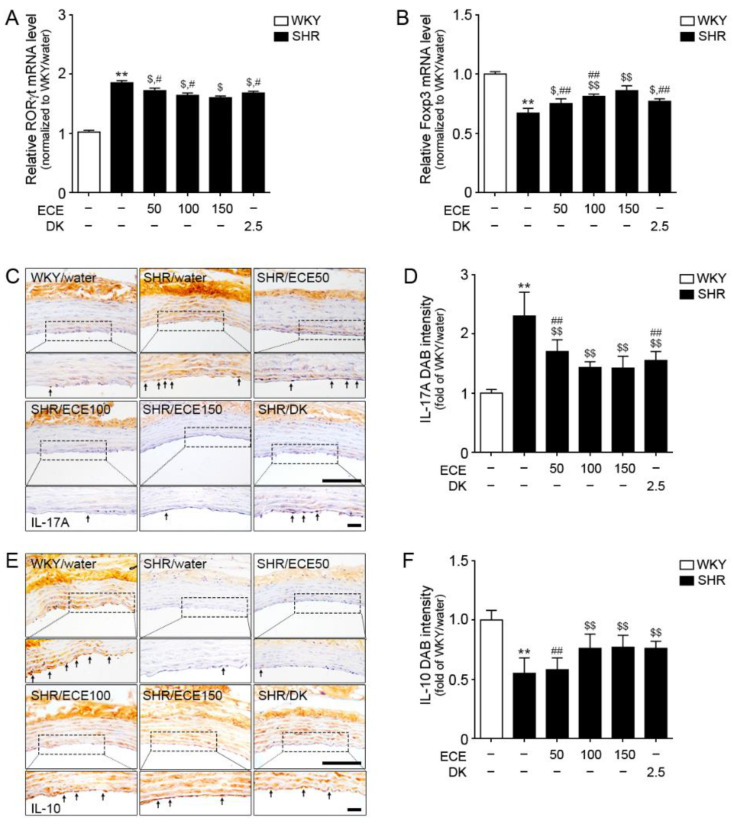
Regulatory effects of DK and ECE on Th17 and Treg cell levels in the aorta of SHRs. (**A**) In the aorta tissue, RORγt mRNA levels were increased by SHR/water. The addition of ECE and DK decreased the RORγt and Foxp3 mRNA levels. (**B**) Foxp3 mRNA level in the aorta tissue was decreased following SHR/water and increased following ECE or DK treatment. (**C**,**D**) The IL-17A expression level in aorta tissue was increased following SHR/water and decreased following ECE or DK treatment. (**E**,**F**) The IL-10 expression level in aorta tissue was decreased following SHR/water and increased following ECE or DK treatment. For each of the 6 groups, *n* = 5. Scale bar = 100 µm. **, *p* < 0.01 vs. WKY/water; $, *p* < 0.05 and $$, *p* < 0.01, vs. SHR/water; #, *p* < 0.05 and ##, *p* < 0.01, vs. SHR/ECE150 (Mann–Whitney U test). DK, dieckol; ECE, *Ecklonia cava* extract; Foxp3, forkhead box P3; IL-10, interleukin-10; IL-17A, interleukin-17A; RORγt, retinoic-acid-receptor-related orphan nuclear receptor gamma t; SHR, spontaneously hypertensive rat; WKY, Wistar Kyoto rat.

The mRNA expression of RORγt (a marker of Th17) in the aorta of SHRs was increased compared with that in WKY rats (Figure 3A). However, its level was significantly decreased by the administration of either ECE or DK. The decreasing effect was most prominent in the 150 mg/kg ECE treatment. The mRNA expression level of Foxp3 (a marker of Tregs) in the aorta of SHRs was significantly lower than that in WKY rats (Fig 3B); however, it was significantly increased following the administration of either ECE or DK. The increasing effect was most prominent in the 150 mg/kg ECE treatment.

The expression level of IL-17A in the aorta of SHRs was significantly increased compared with that in WKY rats (Figure 3C,D). It was significantly decreased following the administration of either ECE or DK. The decreasing effect was most prominent in the 100 mg/kg ECE and DK treatments. The expression level of IL-10 in the aorta of SHRs was significantly decreased compared with that in WKY rats (Figure 3E,F), but it was significantly increased following the administration of either ECE or DK. The increasing effect was most prominent in the 100 mg/kg ECE treatment. 

### 3.5. ECE and DK Attenuated the Expression of the IL-17A/NF-κB/IL-6 Pathway in the Aorta of SHRs

**Figure 4:**
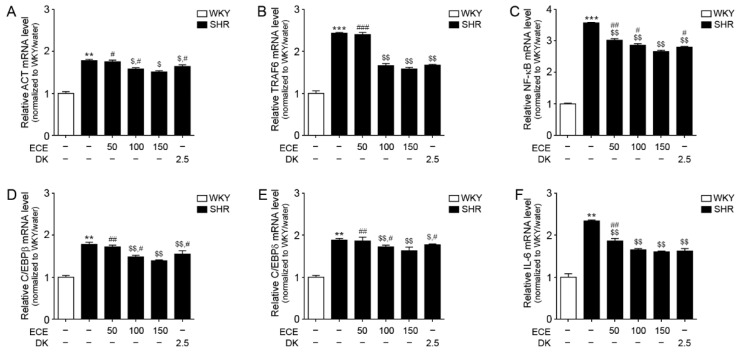
Modulating effects of DK and ECE on the IL-17A/NF-κB/IL-6 pathway in the aorta of SHRs. (**A**–**F**) In aorta tissue, (**A**) ACT, (**B**) TRAF6, (**C**) NF-κB, (**D**) C/EBPβ, (**E**) C/EBPδ, and (**F**) IL-6 mRNA levels were increased by SHR/water. The addition of ECE and DK decreased the ACT, TRAF6, NF-κB, C/EBPβ, C/EBPδ, and IL-6 mRNA levels. For each of the 6 groups, *n* = 5. **, *p* < 0.01 and ***, *p* < 0.001 vs. WKY/water; $, *p* < 0.05 and $$, *p* < 0.01, vs. SHR/water; #, *p* < 0.05 and ##, *p* < 0.01, vs. SHR/ECE150 (Mann–Whitney U test). ACT, NF-κB activator1; C/EBPβ, CCAAT/enhancer-binding protein beta; C/EBPδ, CCAAT/enhancer-binding protein delta; DK, dieckol; ECE, *Ecklonia* *cava* extract; IL-6, interleukin-6; NF-κB, nuclear factor kappa B; SHR, spontaneously hypertensive rat; TRAF6, tumor necrosis factor receptor-associated factor 6; WKY, Wistar Kyoto rat.

The expression levels of ACT and TRAF6, which are essential factors for IL-17A to initiate the upregulation of NF-κB, was significantly higher in the aorta of SHRs than in WKY rats (Figure 4A,B). It was significantly decreased following the administration of the 100 and 150 mg/kg ECE and DK treatments. The decreasing effect on ACT was most prominent in the 150 mg/kg ECE treatment. The decreasing effect on TRAF6 among the 100 and 150 mg/kg ECE and DK treatments was not significantly different.

The expression level of NF-κB in the aorta of SHRs was significantly higher than that in WKY rats (Figure 4C). It was significantly decreased following the administration of either ECE or DK. The decreasing effect was most prominent in the 150 mg/kg ECE treatment.

The expression levels of C/EBPβ and C/EBPδ in the aorta of SHRs were significantly higher than that in WKY rats (Figure 4D,E). It was significantly decreased following the administration of either ECE or DK. The decreasing effect was most prominent in the 150 mg/kg ECE treatment.

The expression levels of IL-6 in the aorta of SHRs were significantly higher than that in WKY rats (Figure 4F). It was significantly decreased following the administration of either ECE or DK. The decreasing effect among the 100 and 150 mg/kg ECE and DK treatments was not significantly different. 

### 3.6. ECE and DK Attenuated Endothelial Cell Dysfunction in the Aorta of SHRs

**Figure 5:**
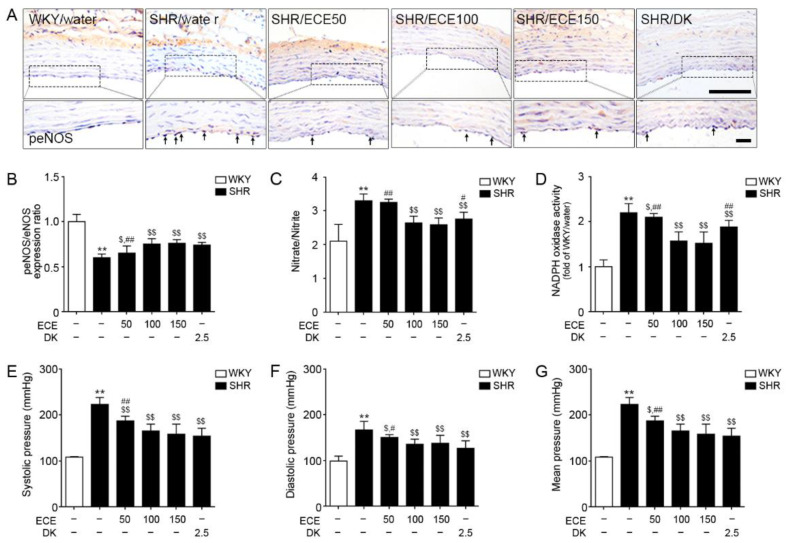
Modulating effects of DK and ECE on endothelial cell dysfunction in the aorta of SHRs. (**A**,**B**) The peNOS/eNOS expression level in aorta tissue was decreased following SHR/water and increased following ECE or DK treatment. (**C**) The nitrate/nitrite in aorta tissue was increased following SHR/water and decreased following ECE or DK treatment. (**D**) The relative NDAPH/NADP^+^ ratio in aorta tissue was increased following SHR/water and decreased following ECE or DK treatment. (**E**,**F**) The systolic BP (**E**), diastolic BP (**F**), and mean BP (**G**) were increased following SHR/water and decreased following ECE or DK treatment. For each of the 6 groups, *n* = 5. Scale bar = 100 µm. **, *p* < 0.01 vs. WKY/water; $, *p* < 0.05 and $$, *p* < 0.01, vs. SHR/water; #, *p* < 0.05 and ##, *p* < 0.01, vs. SHR/ECE150 (Mann–Whitney U test). BP, blood pressure; DAB, 3,3-diaminobenzidine; DK, dieckol; ECE, *Ecklonia* *cava* extract; peNOS, phosphorylated endothelial nitric oxide synthase; SHR, spontaneously hypertensive rat; WKY, Wistar Kyoto rat**.**

The ratio of peNOS/eNOS expression in the aorta of SHRs was significantly lower than that in WKY rats (Figure 5A,B and Figure S3). It was significantly increased following the administration of either ECE or DK. There were no significant differences in these effects among the 100 mg/kg and 150 mg/kg ECE and DK treatments.

The nitrate/nitrite level in the aorta of SHRs was significantly higher than that in WKY rats (Figure 5C). It was significantly decreased following the administration of either ECE or DK. The decreasing effect was most prominent in the 100 and 150 mg/kg ECE treatments. The relative NDAPH/NADP^+^ ratio in the aorta of SHRs was significantly higher than that in WKY rats (Figure 5D). It was significantly decreased following the administration of either ECE or DK. The decreasing effect was most prominent in the 100 and 150 mg/kg ECE treatments.

The systolic blood pressure (BP) of SHRs was significantly higher than that of WKY rats (Figure 5E). It was significantly decreased following the administration of either ECE or DK. The decreasing effect among the 100, 150 mg/kg ECE, and DK treatments was not significantly different. The diastolic BP and mean BP of SHRs were significantly higher than that of WKY rats. Those were significantly decreased following the administration of either ECE or DK. The decreasing effect among the 100, 150 mg/kg ECE, and DK treatments was not significantly different (Figure 5F,G).

## 4. Discussion

Endothelial cells, which make a lining of blood vessels, have prothrombotic, proinflammatory, and proconstrictive actions [43]. Endothelial dysfunction means endothelial cells alter their own phenotype and lose their functions, such as prothrombotic, proinflammatory, and proconstrictive actions [43]. Many studies have shown that increases in systemic oxidative stress and vascular inflammation are related with the pathogenesis of hypertension [44,45]. Increased vascular oxidative stress and vascular inflammation are also main findings of endothelial dysfunction [46]. It is well established that endothelial dysfunction is associated with hypertension [47,48,49,50,51]. Framingham offspring cohort exhibited that the severity of hypertension increases according to increase of endothelial function dysfunction [52]. 

Many studies have shown that gut epithelial cell barriers are associated with endothelial cell dysfunction, which is a major pathophysiological factor underlying hypertension [53]. Recently, many studies have suggested that the connection between changes in the gut epithelial barrier and hypertension involves changes in Th17 cells or the Treg/Th17 cell balance [54,55]. Th17 cells are also involved in changes to the gut epithelial cell barrier by increasing inflammation, and increases in Th17 cells or IL-17A are related with endothelial cell dysfunction [53]. We evaluated whether ECE or DK attenuated the changes in the gut epithelial barrier and Treg/Th17 balance in the intestine and attenuated the changes in the Treg/Th17 balance in the aorta associated with endothelial cell dysfunction through the IL-17A/NF-κB/IL-6 pathway in SHRs.

We evaluated intestinal barrier impairment by measuring the number of goblet cells, thickness of tunica muscularis, and length of villi. It was reported that these changes of intestinal impairment were shown in SHR [56]. Our study showed similar results. SHR exhibited decreasing number of goblet cells and length of villi. Moreover, SHR showed increased thickness of tunica muscularis. It is known that increased muscularis layer is the typical finding of inflammatory intestinal edema [57]. In our study, the mRNA expression level of a marker of Th17 cells was increased and the level of Tregs was decreased in the intestine of SHRs compared with that in WKY rats. The expression level of IL-17A in the intestine of SHRs was significantly increased compared with that in WKY rats. Moreover, the expression level of IL-10 in the intestine of SHRs was significantly decreased compared with that in WKY rats.

SHR is the most widely used animal model for essential hypertension, which the incidence is up to 95% in human hypertension [58]. In addition, many studies showed endothelial cell dysfunction in SHR [59,60]. Nitric oxide (NO), an endothelium-derived relaxation factor, regulates vascular tone, vascular remodeling, platelet aggregation, and vascular smooth muscle cell proliferation [1,6,61]. Nitric oxide synthase (NOS) produces NO from L-arginine, oxygen, and cofactors [5,54]. In the endothelium, NO is produced mainly by eNOS [8,62], so the activity of eNOS is considered as a marker of endothelial cell dysfunction. The level of reactive oxygen species (ROS) in vascular walls is involved in endothelial dysfunction in SHRs [63]. NADPH oxidase is the principal source of ROS in vascular walls, so NADPH oxidase activity has also been used as a marker of endothelial cell dysfunction [53]. The ratio of nitrate/nitrite is another marker of endothelial cell dysfunction and total nitrate/nitrite of SHRs was increased compared with that in WKY controls [62]. Decreasing NO level, which is induced by endothelial dysfunction, elevated vascular resistance and hypertension [49].

Thus, we thought SHR was the proper animal model to evaluate the effect of ECE and DK on endothelial cell dysfunction in hypertension. In addition, the results of our study showed that the expression of Th17 cell/Treg and IL-17A in the intestine of SHR was different from WKY. It suggested that SHR might be the proper model to evaluate the attenuation effect of ECE or DK in the intestine by modulating Th17 cell/Treg balance. 

In our study, ECE or DK showed to be preserved the intestinal barrier in SHR. We also evaluated expression of ZO-1 and occludin in the intestine of SHR. The expression level of ZO-1 and occludin in the intestine of SHRs was significantly decreased compared with that in WKY rats, and it was increased following the administration of ECE or DK. It is known that increased gut permeability is accompanied by decreasing gut junctional proteins in an angiotensin II-induced hypertension animal model [5]. Prehypertensive SHRs exhibited lower expression of gut junctional proteins even before presenting changes in gut barrier permeability [5].

The administration of ECE or DK significantly decreased the expression level of a marker of Th17 cells and increased the expression of a marker of Treg cells in the intestine of SHRs. The expression level of IL-17A in the intestine of SHRs was also decreased following the administration of ECE or DK. Meanwhile, the expression level of IL-10 in the intestine of SHRs was significantly increased by ECE or DK treatment. It seems that ECE or DK restored the Treg/Th17 balance in the intestine of SHR, decreased the expression of IL-17A, and increased the expression of IL-10.

It is known that SGK1 involves changes of Th17/Treg balance [24]. SGK1 inhibited Foxp3 expression via IL-23 receptor and restrains Treg cell expansion [30]. In our study, the results showed that the expression of SGK1 in the intestine of SHRs was increased compared with that in WKY. Moreover, the expression of SGK1 was significantly decreased by either ECE or DK. The serum level of IL-17A increased in SHR and it was significantly decreased by either ECE or DK. Moreover, the serum level of IL-10 decreased in SHR and it was significantly increased by either ECE or DK. In many human cohort studies, it has been reported that IL-17A serum levels were related to refractory hypertension [53,64]. It is known that Th17 cells in the aorta are increased in SHR, leading to endothelial cell dysfunction [65]. 

In our study, the number of Th17 cells was increased in the aorta of SHRs, but was significantly decreased by either ECE or DK. Moreover, the number of Treg cells was decreased in the aorta of SHRs, but was significantly increased by either ECE or DK. The expression level of IL-17A was increased in the aorta of SHRs, whereas the expression of IL-10 was decreased. These two changes were reversed following the administration of ECE or DK. We also evaluated the IL-17A/NF-κB/IL-6 pathway, which induced endothelial cell dysfunction. IL-6 is considered a biomarker in the development of atherosclerosis and progression of inflammation in atherosclerotic vessels [66,67]. IL-6, a major proinflammatory cytokine, is involved in endothelial cell dysfunction [68].

The binding of IL-17A to IL-17RA requires ACT and TRAF6 to activate NF-κB [14]. NF-κB activates C/EBPβ and C/EBPδ, leading to an increase in the transcription of IL-6 [69]. IL-17A leads to an increase of IL-6 [70,71], and IL-6 promotes the formation of Th17 cells [72]. It has been suggested that there is a positive feedback loop between IL-17A and IL-6 [72]. In our study, ACT and TRAF6 in the aorta of SHR were increased, but were decreased by the administration of ECE or DK. The expression of NF-κB/C/EBPβ and C/EBPδ in the aorta of SHRs was increased, but was decreased by the administration of ECE or DK. Moreover, the expression of IL-6 was increased, but this was decreased by the administration of ECE or DK.

The peNOS/eNOS ratio in the aorta of SHRs was decreased, but it was increased by ECE or DK treatments. The nitrate/nitrite ratio in the aorta of SHRs was increased, but it decreased following ECE or DK. NADPH oxidase activity was increased in the aorta of SHRs, which was decreased by ECE or DK treatment. It appeared that ECE or DK attenuated endothelial cell dysfunction in SHR. The systolic BP, diastolic BP, and mean BP of SHR were decreased by administration of either ECE or DK. It seems that the attenuating effect of ECE or DK on endothelial cell dysfunction lead to decrease BP. Phlorotannins from ECE contains DK, 2,7-phloroglucinol-6,6-bieckol (PHB), pyrogallol-phloroglucinol-6,6-bieckol (PPB), and phlorofucofuroeckol-A (PFFA) [33,73,74]. In our previous study, PPB also showed effect on attenuating endothelial cell dysfunction [33,35]. Thus, it is possible that ECE showed more prominent effect on decreasing endothelial cell dysfunction by synergistic effect among DK and other phlorotannins than by a single treatment of DK.

ECE contained 2% DK [33]; thus, 150 mg of ECE contained 3 mg of DK. This means that 150 mg/kg of ECE contained more DK than 2.5 mg/kg of DK, which we used in treatment. The attenuating effect of endothelial cell dysfunction among 100 and 150 mg/kg of ECE and 2.5 mg/kg of DK were not significantly different. Thus, we thought that 2.5 mg/kg of DK would have more advantage than 100 and 150 mg/kg of ECE as the treatment dosage.

## 5. Conclusions

Gut health, especially the balance of Treg/Th17 cells, is important for avoiding endothelial cell dysfunction, which is a major pathophysiological factor underlying various cardiovascular diseases including hypertension and atherosclerosis. Our study suggested that ECE or DK could modulate the Treg/Th17 balance in both intestine and aorta, and decrease endothelial cell dysfunction by decreasing the activity of the IL-17A/NF-κB/IL-6 pathway. The results suggested that DK could be potentially used as a therapeutic agent for endothelial dysfunction by modulating gut health. 

## Data Availability

All data is contained within the article.

## Supplementary Materials

The following are available online at https://www.mdpi.com/2076-3921/10/2/298/s1. Table S1: List of primers for qRT-PCR; Table S2: List of antibodies for DAB staining; Figure S1. Regulatory effects of DK and ECE on the destruction of gut barrier junction in the intestine of SHR; Figure S2. Regulatory effects of DK and ECE on IL-17A and Il-10 expression level in the serum of SHR; Figure S3. Regulatory effects of DK and ECE on eNOS expression level in the aorta of SHR.
