# Supplementary material for: Attenuating Effects of Dieckol on Endothelial Cell Dysfunction via Modulation of Th17/Treg Balance in the Intestine and Aorta of Spontaneously Hypertensive Rats
Source: Antioxidants (Basel). 2021 Feb 16;10(2):298. doi: 10.3390/antiox10020298 (PMC7920082; doi:10.3390/antiox10020298)
Supplement: Supplementary file 1 [file antioxidants-10-00298-s001.pdf]

Table S1. List of primers for qRT-PCR

Gene		Primers	Accession number
β-actin	Forward	5'-CCGTAAAGACCTCTATGCCAAC-3′	[Genebank : NM_031144.3]
	Reverse	5'-GGCAGTAATCTCCTTCTGCATC-3′	
RORγt	Forward	5'-TCCAAGAAAAGAGGAGAGTGGA-3′	[Genebank : XM_017591313.2]
	Reverse	5'-GTCGATGAGTCTTGCAGAGATG-3′	
Foxp3	Forward	5'-CTACAGTGCCCCTAGTCATGGT-3′	[Genebank : NM_001108250.1]
	Reverse	5'-TGCATAAAGTGTGGTCTGTCCT-3′	
SGK1	Forward	5'-AGTTTTGCAGAAGAAAGCCATC-3′	[Genebank : NM_001193568.1]
	Reverse	5'-GAAAGGGTGCTTCACATTCTTC-3′	
ACT1	Forward	5'-TGAAGTGTGTGAACTTCCTGCT-3′	[Genebank : NM_001044248.1]
	Reverse	5'-TATCAATACCCCGGATTCTGTC-3′	
TRAF6	Forward	5'-AGATGGAAGCACAGCAGTGTAA-3′	[Genebank : NM_ 001107754.2]
	Reverse	5'-CCTCTTCTTGGGATTTCAAGTG-3′	
NF-κB	Forward	5'-CGGATTTGGAAACTAGTGAACC-3′	[Genebank : NM _001276711.1]
	Reverse	5'-CTTCTGGCGTTTCCTCTGTACT-3′	
C/EBPβ	Forward	5'-ACTTCAGCCCCTACCTGGAG-3′	[Genebank : NM_001301715.1]
	Reverse	5'-GAAGAGGTCGGAAAGGAAGTC-3′	
C/EBPδ	Forward	5'-GACTTCAGCGCCTACATTGATT-3′	[Genebank : NM_013154.2]
	Reverse	5'-TTGTGATTGCTGTTGAAGAGGT-3′	
IL-6	Forward	5'-ATGTTCTCAGGGAGATCTTGGA-3′	[Genebank : NM_02589.2]
	Reverse	5'-GCATCATCGCTGTTCATACAAT-3′	
Occludin	Forward	5'-CCCAGGCTTCTGGATCTATGTA-3′	[Genebank : NM_031329.3]
	Reverse	5'-ACCAGTGCCTCCAGGAGTATAA-3′	

Table S2. List of antibodies for DAB staining

Antigen (host)	Company	Cat. No	Dilution rate
IL-17A (rabbit)	Santa cruz	sc-374218	1:100
IL-10 (mouse)	Santa cruz	sc-8438	1:100
Zo-1 (rabbit)	Bioss	BS-1329R	1:250
peNOS (rabbit)	Santa cruz	sc-136519	1:50
eNOS (rabbit)	Invitrogen	PA3-031A	1:250

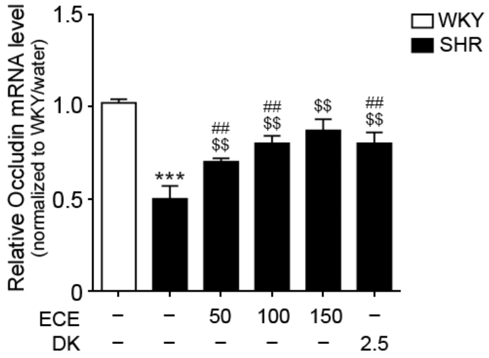

Figure S1. Regulatory effects of DK and ECE on the destruction of gut barrier junction in the intestine of SHR. (A, B) Occludin expression level in intestinal tissue was decreased by SHR/water and increased by ECE or DK treatment. N=5 for each of the 6 groups. ***, $p < 0.001$, vs. WKY/water; $$, $p < 0.01$, vs. SHR/water; ##, $p < 0.01$, vs. SHR/ECE150 (Mann Whitney U test). ECE, *Ecklonia cava* extract; DK, diekol; WKY, wistar Kyoto; SHR, spontaneously hypertensive rat.

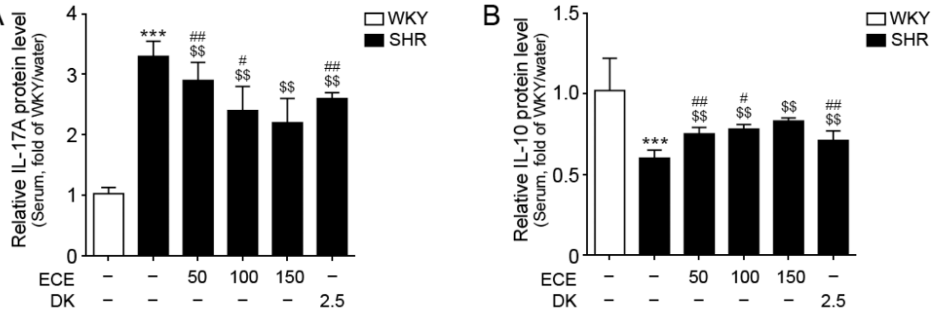

Figure S2. Regulatory effects of DK and ECE on IL-17A and Il-10 expression level in the serum of SHR. (A) In the serum, IL-17A expression levels were increased by SHR/water. Addition of ECE and DK decreased the IL-17A level. (B) IL-10 expression serum levels were decreased by SHR/water. Addition of ECE and DK increased the IL-10 level. N=5 for each of the 6 groups. ***, $p < 0.001$, vs. WKY/water; $$, $p < 0.01$, vs. SHR/water; #, $p < 0.05$ and ##, $p < 0.01$, vs. SHR/ECE150 (Mann Whitney U test). IL-17A, interleukin-17A; IL-10, interleukin-10; ECE, *Ecklonia cava* extract; DK, diekol; WKY, wistar Kyoto; SHR, spontaneously hypertensive rat.

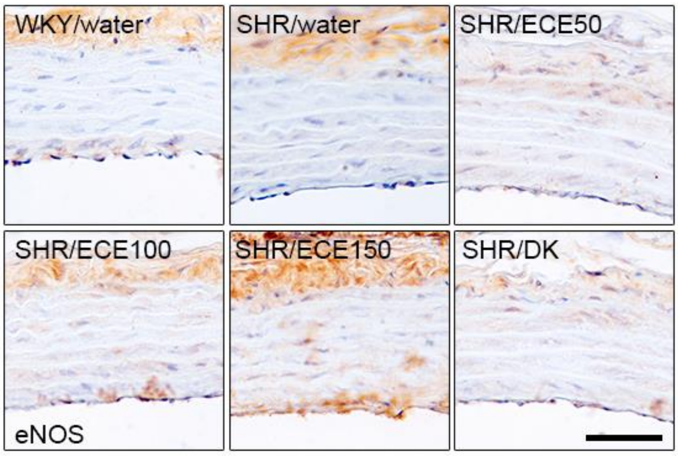

Figure S3. Regulatory effects of DK and ECE on eNOS expression level in the aorta of SHR. In the aorta, eNOS expression levels were decreased by SHR/water. Addition of ECE and DK decreased the eNOS level. N=5 for each of the 6 groups. Scale bar = 100 μm. ECE, *Ecklonia cava* extract; DK, diekol; WKY, wistar Kyoto; SHR, spontaneously hypertensive rat.
